# Autonomic dysfunction in multiple system atrophy: from pathophysiology to clinical manifestations

**DOI:** 10.1080/07853890.2025.2488111

**Published:** 2025-04-08

**Authors:** Yuqi Luo, Nan Yang, Wanlin Yang, Baoling Chen, Shuzhen Zhu, YihRu Wu, Qing Wang

**Affiliations:** a Department of Neurology, Zhujiang Hospital of Southern Medical University, Guangzhou, China; b Department of Neurology and Rehabilitation, Zhongshan Hospital of Traditional Chinese Medicine, Zhongshan, China; c Department of Neurology, Chang Gung Memorial Hospital Linkou Medical Center and College of Medicine, Chang-Gung University, Taoyuan, Taiwan

**Keywords:** Multiple system atrophy, central autonomic network, autonomic dysfunction, pathophysiology, clinical manifestations

## Abstract

**Introduction:**

Multiple system atrophy (MSA) is a sporadic, fatal, and rapidly progressive neurodegenerative disease of unknown etiology, pathologically characterized by the presence of α-synuclein (α-syn) immunoreactive cytoplasmic inclusions in oligodendrocytes. The deposition of α-synuclein in highly interconnected neuronal networks with different neurochemistry properties in different regions of the cortex, diencephalon, brain stem and spinal cord leads to early onset and extensive autonomic dysfunction in MSA. Mainly affected areas include the hypothalamus, pons, raphe nucleus, locus coeruleus, arcuate nucleus, dorsal vagus nucleus, fuzzy nucleus, the thoracolumbar middle lateral column and Onuf’s nucleus of the spinal cord. Clinical manifestations include orthostatic hypotension, incomplete bladder emptying, erectile dysfunction, and constipation.

**Discussion:**

In this review, we aim to discuss and summarize the clinicopathological correlation of MSA autonomic dysfunction, and focus on the pathophysiological mechanisms of various autonomic dysfunction, from neural control networks under normal physiological conditions to specific pathological involvement structures in MSA. In addition, we also elaborated on the corresponding clinical manifestations caused by various pathological structures.

**Conclusions:**

In summary, the autonomic dysfunction of MSA involves the comprehensive control of cardiovascular, urinary, reproductive, and gastrointestinal functions by the autonomic nervous network in the central nervous system (CNS). The currently summarized physiology and pathophysiology of MSA have not been fully clarified. Further and deeper studies are needed to elucidate the relationship between pathogenesis and clinical manifestations of MSA.

## KEY POINTS

- The α-synuclein in the autonomic nervous system leads to early onset and severe autonomic dysfunction in MSA.
- The autonomic dysfunction of MSA covers all aspects of autonomic control, including cardiovascular dysfunction, urinary dysfunction, sexual dysfunction, and gastrointestinal dysfunction.
- The specific pathological structures involved in MSA leads to corresponding clinical manifestations.

## 1. Introduction

Multiple system atrophy (MSA) is a fatal neurodegenerative disease of unknown etiology. The average life expectancy of patients after diagnosis is 6.2–10 years, and unfortunately, there is currently no available treatment that can modify the disease. MSA affects both sexes and has a prevalence ranging from 1.9 to 4.4 per 100,000, and an incidence of 3 per 100,000 per year. MSA usually occurs in middle age (54–61 years on average) [1,2]. MSA is mainly divided into two phenotypes on the basis of clinical manifestations: multiple system atrophy with predominant cerebellar ataxia (MSA-C) and multiple system atrophy with predominant parkinsonian symptoms (MSA-P). The cerebellar symptoms of MSA include scanning dysarthria, limb motor and gait ataxia and cerebellar oculomotor dysfunction. The parkinsonian symptoms of MSA are characterized by bradykinesia, stiffness, postural instability, speech weakness and tremor, and generally poor response to levodopa [2–10]. However, regardless of the phenotype, the vast majority of MSA patients will develop autonomic dysfunction, which may occur earlier than the motor symptoms. Autonomic dysfunctions include orthostatic hypotension (OH), supine hypertension, detrusor muscle overactivity, incomplete bladder emptying, sexual desire deficiency, erectile dysfunction, delayed gastric emptying and constipation. The median period from the initial onset to the presence of combined motor and autonomic dysfunctions is reported to be 2 years [11,12]. Other nonmotor symptoms include snoring, rapid-eye-movement sleep behavior disorder, obstructive sleep apnea, laryngeal stridor and restless leg syndrome [2,13,14]. The adverse prognostic factors related to a short survival period are early onset of OH, bradykinesia, inspiratory wheezing and the parkinsonian variant [15].

The diverse clinical manifestations of MSA are due to the widespread distribution of pathological markers-glial cytoplasmic inclusions (GCIs) in the CNS, especially in the pyramidal, extrapyramidal, cortical cerebellar and preganglionic autonomic nervous systems. GCIs, with a diameter of approximately 5–20 µm, generally exist in oligodendrocytes near or around the nucleus; they can be triangular, sickle shaped, half-moon shaped, elliptical, conical or flame shaped; and they show α-synuclein (α-syn) immunoreactivity [16–19]. Additionally, α-syn immunoreactive inclusions can also be found in the glial nucleus, neuronal cytoplasm and neuronal nucleus. The α-syn immunoreactive cell inclusions, together with selective neuronal loss, axonal degeneration, glial hyperplasia and a pale myelin sheath, constitute the main histopathological features of MSA [15,20]. The presence of these pathological changes in the dorsolateral putamen and caudate nucleus lead to the clinical manifestations of MSA-P, such as retardation and stiffness. The presence of these pathological changes in the pontine basis, inferior olivary nucleus, cerebellar dentate nucleus, cerebellar vermis, and Purkinje cells lead to the clinical manifestations of MSA-C, such as ataxia and dysarthria [21,22]. Pathologically, MSA-P is related to striato-nigral degeneration, while MSA-C is related to olivopontocerebellar atrophy [23]. The study of brain slices of MSA patients after death found that the cerebellar subcortical white matter and cerebellar brainstem projections may be the earliest pathological lesions of α-syn in MSA-C [24]. While, another study of striato-nigral degeneration MSA cases showed that the most severe α-syn pathology was first observed in the striatum and lentiform nucleus [25]. However, with the extension of the course of MSA, it usually shows the overlapping pattern of α-syn pathology. GCIs and the resulting neurodegeneration are involving not only the striatum and olivopontocerebellar atrophy system, but also the cortical area, autonomic nerve and motor nucleus in the brain stem, spinal cord, preganglionic autonomic nervous structure and peripheral nervous system [26,27]. The extensive involvement of the central autonomic neural network, including the hypothalamus, ventrolateral part of the intermediate reticular formation, pons, raphe nucleus, locus coeruleus, arcuate nucleus, dorsal vagus nucleus, fuzzy nucleus, thoracolumbar middle lateral column and Onuf’s nucleus of the spinal cord, leads to the failure of autonomic function in MSA [15,22,28]. A clinical study[29] comparing non-motor symptoms among patients with MSA, Parkinson’s Disease (PD), and progressive supranuclear palsy (PSP) with the NMS Scale (NMSS) system. The results showed that the total NMSS score of the MSA group was higher than that of the PD group and PSP group. REM-sleep behavior disorder (RBD), constipation, problems having sex, and loss of sexual interest preceded the motor symptoms onset of MSA. However, it is not clear how these α-syn immunoreactive GCIs are produced and lead to multi-regional involvement and neuronal degeneration. Sekiya et al. [30] measured the distribution of α-syn oligomer in MSA patients’ brain, and extended the analysis area to the neocortex and hippocampus, as well as the striatum and brain stem. The results showed that oligomers were widely distributed and accumulated in neurons and oligodendrocytes of the neocortex in MSA. The accumulation of protein oligomer may be the early pathological change of MSA.

However, the cellular localization, cytotoxicity, and seeding activity of α-syn aggregates in PD [10,31–33] and MSA are different. A recent study compared the differences in a-syn oligomers formed in the plasma of MSA and PD patients and found that α-syn proteins incubated in PD and MSA plasma could aggregate into oligomers with significantly different cytotoxic and seeding activities [34]. The main motor and nonmotor clinical manifestations and corresponding involved regions in MSA are shown in Table 1.

**Table 1.** The clinical manifestations and corresponding involved regions in MSA.

	Parkinsonism	Cerebellar symptoms	OH	Urinary dysfunction	Sexual dysfunction	Gastrointestinal dysfunction	Thermoregulation disorder	RBD	Respiratory dysfunction
Basal ganglia	+			+		+			
PVN			+		+		+		+
MPOA					+		+		
PAG			+	+	+			+	
PMC				+	+				
PCC				+					
LC			+	+				+	
LDTN								+	+
PPTN								+	+
Medullary raphe nucleus			+	+		+	+		
VLM			+				+		+
A5 neurons			+						
Kölliker–Fuse nucleus									+
Pre-BÖtzinger complex									+
DMV				+		+			
Nucleus ambiguus									+
Cerebellum		+		+					
Inferior olives		+							
Arcuate nucleus									+
IML			+	+	+	+	+		
Onuf’s nucleus				+	+	+			^a^

^a^ OH: orthostatic hypotension; RBD: rapid-eye-movement sleep behaviour disorder; PVN: paraventricular nucleus; MPOA: medial preoptic area; PAG: periaqueductal gray; PMC: pontine micturition center; PCC: pontine continence center; LC: locus ceruleus; LDTN: laterodorsal tegmental nucleus; PPTN: pedunculopontine tegmental nucleus; VLM: ventrolateral medulla; DMV: dorsal motor nucleus of vagus; IML: intermediolateral cell column of thoracolumbar spinal cord.

Due to the uncertainty of the pathogenesis of MSA, less neuroprotective or rehabilitation programs targeting major symptoms and quality of life has been developed to prevent or reverse the progression of this destructive disease. Therefore, the current therapeutic interventions for autonomic dysfunction in MSA are still aimed at improving OH, erectile dysfunction, gastrointestinal disorders and other symptoms [35]. However, at present, some therapeutic experiences of MSA come from PD patients, and most treatments have not been proved to be effective for MSA, which may be due to the different pathological mechanisms of autonomic nervous dysfunction between MSA and PD [33,34]. Therefore, this review introduces and summarizes the pathophysiological mechanism and corresponding clinical manifestations of MSA autonomic dysfunction, which provides a reference for further study on the pathogenesis of MSA and the treatment of autonomic nervous dysfunction.

## 2. Autonomic dysfunction in MSA

The extensive involvement of the autonomic neural network leads to the early onset and typical autonomic dysfunction of MSA. Typically, affected areas are the paraventricular hypothalamus (PH), locus coeruleus (LC), ventrolateral tegmental nucleus, periaqueductal gray (PAG), dorsal vagal nucleus, ventrolateral nucleus ambiguous, C1 neurons in the rostral ventrolateral medulla (RVLM), A1 neurons in the caudal ventrolateral medulla (CVLM), serotonergic neurons in the raphe, and neurons in the pontine micturition area. The main affected areas of the spinal cord are the intermediolateral columns of the thoracolumbar segment and Onuf’s nucleus [36]. Other central autonomic structures involved in MSA include the Edinger-Westphal nucleus and medullary arcuate nucleus [37]. Pathological changes in different areas can lead to varying degrees of sympathetic and parasympathetic reflex damage. The attenuation of sympathetic noradrenergic reflex activation leads to OH and postprandial hypotension. Of note, Gilman’s criteria is the common diagnosis of OH in MSA [23].

Recently, some clinical studies have verified the new standards for the Movement Disorder Society Criteria of 2022 [38]. A retrospective study in autopsy-confirmed patients estimated the diagnostic accuracy of the clinical diagnostic criteria for MSA. The results found that the International Parkinson and Movement Disorder Society multiple system atrophy diagnostic criteria showed consistently high specificity and low to moderate sensitivity throughout the disease course [39]. Sun et al. retrospectively reviewed 73 MSA patients in China, to compare the differences between the 2008 and 2022 criteria for MSA. The results showed that approximately 78.7% of the category of probable patients in the 2008 statement can be categorized as clinically established MSA in the 2022 MDS criteria and five patients with non-supporting features in the 2008 criteria can be diagnosed as clinically probable MSA in the MDS criteria, emphasizing the importance of supportive and imaging features in the diagnosis of MSA [40]. A clinical study involving 587 MSA patients assessed and compared the diagnostic utility of the 2022 movement disorder society (MDS) MSA criteria with the 2008 MSA criteria. And the results found that the sensitivity of the MDS MSA criteria (93.2%, 95% CI = 90.5%–95.2%) was significantly higher than that of the 2008 MSA criteria, exhibiting a good diagnostic utility for MSA [41].

Symptoms and signs of parasympathetic reflex failure include baroreflex cardiac vagal failure, constipation caused by intestinal peristalsis, and urinary incontinence and retention caused by decreased bladder tone. The degeneration of the brainstem and cerebellum can lead to abnormal respiration, repeated inspiration, dysarthria, dysphagia and sleep disorders, increasing the sensitivity of food inhalation and leading to asphyxia or aspiration pneumonia [42,43]. In addition, abnormal circadian rhythms of neurohormones often occur in MSA patients, which may be related to the pathological participation of autonomic neurons in the suprachiasmatic nucleus (SCN) and paraventricular nucleus (PVN) [44]. Other autonomic dysfunctions may include heat and cold intolerance, flushing, adiapneustia and limb cyanosis [43]. Overall, the autonomic dysfunction of MSA involves the comprehensive control of cardiovascular, respiratory, urinary, reproductive, and gastrointestinal functions by the autonomic nervous system in CNS [45].

Next, starting from the anatomy of the autonomic nervous system, then to the pathophysiological ­pathogenesis, and finally to the corresponding clinical symptoms, we will discuss the autonomic nervous ­dysfunction of MSA from four aspects: cardiovascular dysfunction, urinary dysfunction, sexual dysfunction and gastrointestinal dysfunction.

### 2.1. Cardiovascular dysfunction

#### 2.1.1. Normal baroreflex control

Changes in sitting and lying posture will lead to the redistribution of blood in the vessels and alternations in blood pressure, while carotid sinus and aortic arch baroreceptors can compensate for the decrease in blood pressure by regulating the activation of sympathetic nerves. In addition, the activation of the renin angiotensin aldosterone system also helps maintain arterial blood pressure [46–48].

The specific mechanism of pressure reflection is as follows. When blood pressure drops, the baroreceptor signal is transmitted from the neurons of the nucleus tractus solitarius (NTS) to the neurons of the CVLM/intermediate ventrolateral medulla (IVLM) through direct projection [49]. In addition, the unloading of baroreceptors and cardiac receptors caused by hypovolemia and hypotension liberates magnocellular arginine vasopressin (AVP) neurons from the tension inhibition control caused by these receptors and leads to the release of AVP [50]. In short, the neural core network is composed of the NTS, AVP-secreting magnocellular neurons in the hypothalamus, C1 neurons in the RVLM, and the intermediolateral columns of the spinal cord, which control sympathetic nerve efferents [51].

**Figure 1.**
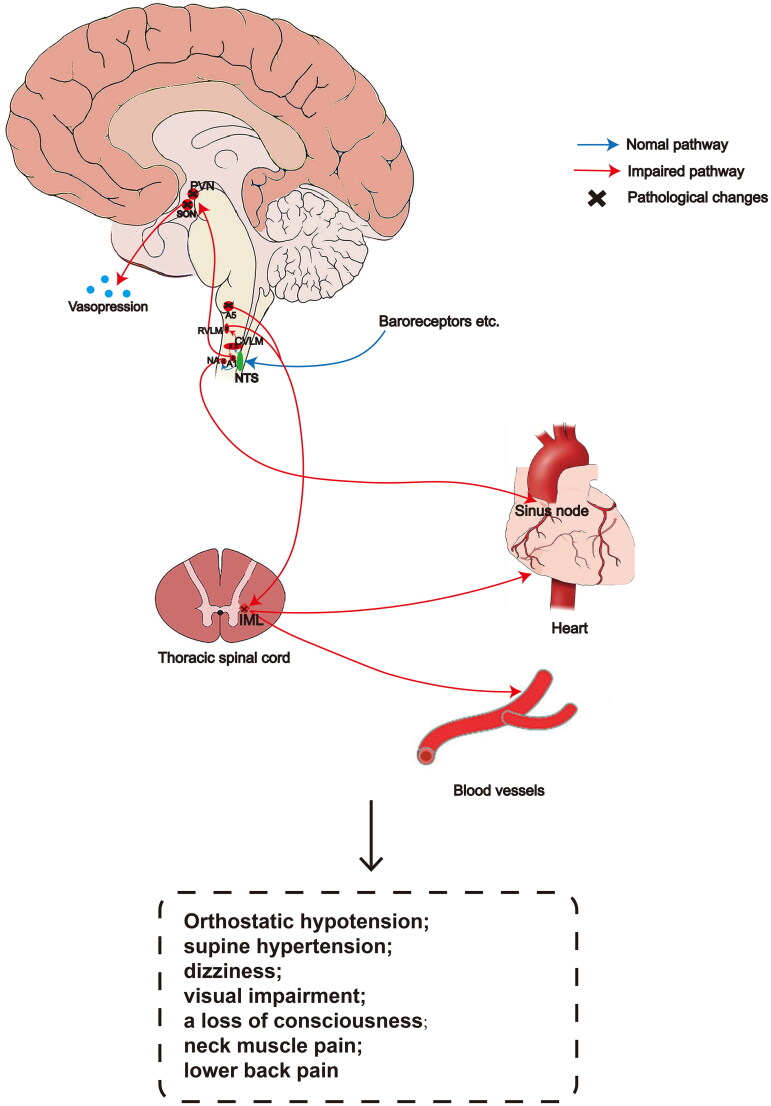
A schematic diagram of pressure reflection in MSA. The decrease of arterial pressure leads to a decline of afferent impulse transmitted from mechanical receptors to the dorsomedial NTS of the medulla oblongata through the glossopharyngeal nerve and vagus nerve, and then mediates the increase of sympathetic efferent activity through the excitatory pathway of the CVLM and the inhibitory pathway of the RVLM. The projection of A1 noradrenergic cell group in the ventrolateral medulla oblongata activates vasopressin synthesis neurons in the large cell parts of hypothalamic PVN and SON, resulting in the release of vasopressin. The pathological changes eventually lead to cardiovascular dysfunction. NTS: nucleus of the solitary tract, CVLM: caudal ventrolateral medulla, RVLM: rostral ventrolateral medulla, NA: nucleus ambiguous, PVN: paraventricular nucleus, SON: supraoptic nucleus, IML: intermediolateral cell column.

The final sympathetic efferent is mediated by the postganglionic neurons of the sympathetic chain synapses of the sympathetic preganglionic nerves (T1 to L2) of the spinal cord intermediolateral cell column (IML). These postganglionic sympathetic nerves form a nerve plexus located in the adventitia layer of blood vessels [52]. A brief schematic of the baroreflex is presented in Figure 1. In addition to the aforementioned baroreflex, nitric oxide (NO), venous atrial reflex and myogenic response can also compensate for a decrease in blood pressure by altering local vascular activity [53].

#### 2.1.2. Neurodegenerative changes and corresponding clinical manifestations in MSA

Intriguingly, in MSA, orthostatic exercise does not increase sympathetic activity, which is mainly manifested by insufficient increases in plasma norepinephrine levels and impaired AVP reflex release. There is obvious hypothalamic denervation in MSA, which may be caused by the loss of ascending catecholaminergic neurons projecting to the hypothalamus and can explain three typical findings, i.e. the lack of vasopressin reflex release for orthostatic hypotension, the impairment of the suprarenal corticosteroid response to hypoglycemia and the impairment of the growth hormone response to the α2-agonist clonidine [45]. Specifically, the impairment of vasopressin reflex release in response to hypotension or hypovolemia is caused by the degeneration of A1 neurons in the CVLM. In addition, the accumulation of α-syn in SCN neurons that synthesize vasopressin may lead to abnormal circadian rhythms and an increase nocturnal urine excretion [54–57].

In addition to the above affected areas related to impaired AVP reflex release, pathological changes in the PAG, locus coeruleus, RVLM, CVLM and spinal cord IML cause insufficient increases in plasma norepinephrine. The lateral PAG column mediates the sympathetic excitatory response through its projection to the RVLM. The loss of cells in this column may lead to the impairment of sympathetic cardiovascular control in MSA [58]. Meanwhile, an autopsy revealed that the loss of tyrosine hydroxylase immunoreactivity in the spinal cord RVLM, CVLM and IML of MSA patients was consistent with the diffuse loss of catecholaminergic cells in the main medullary source of sympathetic preganglionic neurons descending projection [59]. Among them, C1 neurons in the RVLM provide a tonic sympathetic drive for blood vessels, and their loss is closely related to OH in MSA patients [54]. In addition, the A5 neurons of the ventrolateral pontine, serotonergic neurons of the ventromedial medulla, nucleus raphe obscurus (ROb) and raphe pallidus (RPa) receive input from C1 neurons and are widely involved in MSA [59]. The above findings emphasize that although the loss of preganglionic sympathetic neurons in the IML is classically considered to be the cause of OH in MSA patients, the injury of the supraspinal mechanism is also involved in the pathogenesis of OH in MSA [60].

Approximately 75% of patients with MSA will experience OH symptoms [61]. Orthostatic hypotension is common in people over 75 years of age and aging itself can lead to an OH tendency of reduced cardiac output, bradycardia, and decreased baroreceptor sensitivity [62,63]. A decrease in blood pressure can lead to a decline in organ perfusion, such as in the brain and neck, dizziness, visual impairment, a loss of consciousness, neck muscle pain (‘coat hanger’ pain) and lower back pain [52]. In addition, orthostatic dyspnea caused by insufficient pulmonary apex perfusion during ventilation and angina pectoris caused by impaired myocardial perfusion may also occur [64]. Nonspecific symptoms include drowsiness, fatigue, weakness, and falls. Patients with orthostatic hypotension usually have the lowest blood pressure and more severe symptoms in the morning, which may be related to the decrease in extracellular fluid caused by nocturnal polyuria. In addition to this time point in the morning, many factors such as the speed of posture change, warm environment, food and alcohol intake may lead to further aggravation of hypotension. Moreover, drugs such as diuretics, dehydration and antihypertensive drugs may also aggravate OH symptoms in MSA patients [65,66].

MSA patients with OH are also prone to postprandial hypotension. Research has found that the degree of blood pressure decrease is related to the proportion of various components in food. A diet with a high proportion of glucose is more significant in inducing postprandial hypotension than a diet with a high proportion of fat. In contrast, the higher the protein content, the smaller the change in blood pressure [67]. The specific symptoms of postprandial hypotension may include visual impairment, dizziness, presyncope and syncope similar to OH [52].

It should be noted that besides orthostatic hypotension, supine hypertension is another common cardiovascular dysfunction in MSA. The low-dose ganglion blocker trimetazidine or adrenoceptor antagonist α-phentolamine can significantly reduce blood pressure in patients with MSA, indicating that residual sympathetic tension may be the cause of supine hypertension in MSA patients [68]. Other clinical symptoms can also be well explained by the postganglionic residual sympathetic nerve activity model in MSA. For example, many MSA patients have cold, dark, purplish red hands and poor capillary filling [69]. In addition to the residual sympathetic tone, frequent OH may continue to activate the renin-angiotensin system, leading to an increase in blood pressure [70]. Supine hypertension with autonomic nervous failure may be severe and even lead to terminal organ damage, such as impaired renal function and left ventricular hypertrophy. Stress diuresis caused by supine hypertension increases nocturia and worsens morning OH [46]. In addition, clinical reports of acute events such as papilledema, stroke, cerebral hemorrhage and heart failure due to supine hypertension also occur occasionally [71].

In addition to the above clinical manifestations, other autonomic cardiovascular diseases, such as low RR variability and denervation hypersensitivity of blood vessels and the heart, can also be observed in MSA patients [59]. A prospective study on phenoconversion of pure autonomic failure patients found that subjects reporting deterioration of handwriting were more likely to phenoconvert to PD, difficulty handling utensils were more likely to DLB and patients with a younger age of pure autonomic failure onset, preserved olfaction, anhidrosis and severe urinary problems were more likely to MSA [72]. Postganglionic sympathetic degeneration is a characteristic feature of idiopathic PD, whereas patients with MSA-P exhibit preganglionic abnormalities. A recent study used ^123^I-MIBG SPECT-CT and chest computed tomography to differentiate PD from MSA [73].

### 2.2. Urinary dysfunction

#### 2.2.1. Normal lower urinary tract control

**Figure 2.**
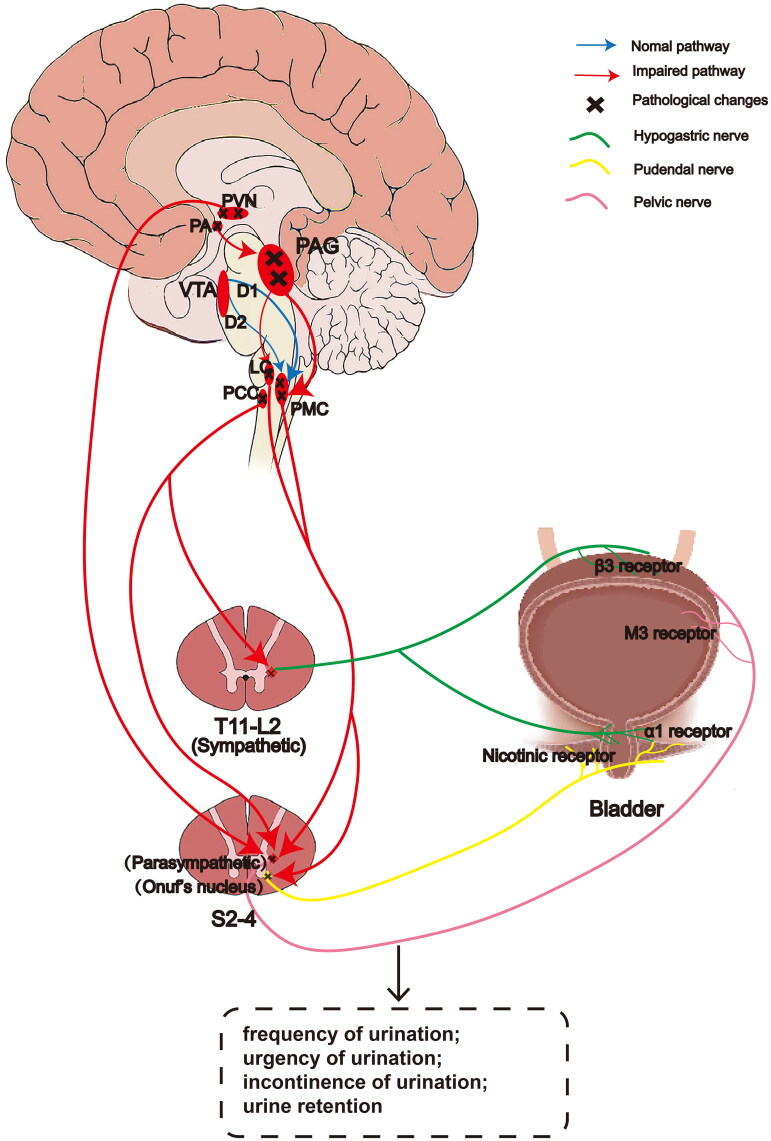
Simplified diagram for the main neural circuits associated with bladder control in MSA. Urine storage is thought to be promoted by the brain, especially the PCC. Hypothalamus, cerebellum, basal ganglia and frontal cortex further promote storage function. Micturition is initiated by the hypothalamus and prefrontal cortex involved the midbrain PAG, PMC and LC. VTA is involved in micturition control through D1 and D2 receptors. Parasympathetic nerve fibers come from S2–S4 nerve roots which travel in the pelvic nerve and innervate the bladder detrusor muscle through the pelvic plexus. Sympathetic fibers from the T11–L2 segments pass *via* the inferior mesenteric plexus and travel in the hypogastric nerve to innervate the ureter and detrusor smooth muscle. The somatic innervation of the external urethral sphincter is through the pudendal nerve and originates from the Onuf’s nucleus of S2–S4. The pathological changes eventually lead to urinary dysfunction. PVN: paraventricular nucleus; PA: preoptic area; VTA: ventral tegmental area; D1: dopamine D1 receptors; D2: dopamine D2 receptors; PAG: periaqueductal gray area; PMC: pontine micturition center; LC: locus ceruleus; PCC: pontine continence center; T: thoracic; L: lumbar; S: sacral.

The lower urinary tract (LUT) is mainly composed of the bladder and urethra, which are innervated by sympathetic, parasympathetic and somatic nerves. Sympathetic norepinephrine fibers innervate the bladder through adrenergic β3 receptors, leading to bladder relaxation. At the same time, they innervate the urethra *via* adrenergic α-1 A/D receptors and mediate urethral contraction. Parasympathetic cholinergic fibers mediate bladder contraction through muscarinic M2, 3 receptors. The somatic nerves send out cholinergic fibers, which control the urethra through nicotinic receptors to make it contract. In addition to the peripheral sympathetic, parasympathetic and somatic nerves, the complete neural control of the LUT involves almost all nervous systems, with the pons being the most important control center. In addition, the complete connection between the pontine and sacral medulla is also the basis for achieving complete neural control of the LUT. This is different from postural hypotension, where the lesion is mainly located below the medullary circulation center [12,74]. The two main functions of the LUT, urine storage under low pressure and regular and complete voluntary urination, are closely related to complete neural control [75]. A brief schematic of the neural control of the LUT is shown in Figure 2.

The ‘voiding mode’ refers to the condition in which the bladder sphincter is relaxed, the detrusor is contracted, and urination is allowed [76]. The main afferent nerve is the pelvic nerve, which transmits signals of bladder filling to the sacral spinal cord. Subsequently, when the secondary neurons in the spinal cord transmit urination information to the central nervous system, many regions activate and maintain urination. The most important area for urination is the M region in the dorsal pontine, which is equivalent to the classic Barrington nucleus or pontine micturition center (PMC) described in experimental animals, and it can release corticotropin releasing factor, glutamate and other cotransmitters. Neurons in this region directly send excitatory projections to the sacral parasympathetic nucleus (the intermediolateral cell group), while projecting fibers containing γ-aminobutyric acid and glycine directly inhibit Onuf’s nucleus [77,78]. Besides, the potential target of PMC projection also includes the lateral part of the dorsal motor nucleus of the vagus (DMV), which indicates other functions in addition to urination [79]. The transition from ‘storage mode’ to ‘voiding mode’ is mediated by PAG. The lateral PAG is the main target for sacral spinal cord input and maintains a specific projection to the PMC [77]. A study of positron emission tomography (PET) scans demonstrated that PAG activity increased with bladder filling [80]. When the amount of urine in the bladder reaches the standard for starting urination, PAG neurons activate the premotor interneurons in the M region to begin urination. In summary, the PAG is the conversion center from storage to voiding [78].

In addition to the PMC and PAG, other regions of the cortex, pons and medulla oblongata are also involved in controlling urination. The basal ganglia mainly inhibit urination [81]. Dopaminergic neurons derived from the ventral tegmental area (VTA) control the urination reflex in a biphasic manner [82]. In addition, serotonergic neurons in the raphe nucleus also provide downward projections to Onuf’s nucleus, and the PVN also contains projection fibers of autonomic motor neurons and Onuf’s nucleus, thus participating in urination control [77]. At present, the suprabridge innervation of the PMC and PAG is not completely clear, but many studies have proposed some possible regions. Studies have suggested that functional magnetic resonance imaging (fMRI) activities related to urination mainly occur in the bilateral cingulate cortex, bilateral medial frontal cortex, occipital parietal area, insular lobe and parahippocampal gyrus [76]. In all studies, the right inferior frontal gyrus of the prefrontal cortex was active during the storage and urination stages of bladder function and is believed to determine whether to urinate at a specific time or place [78,83].

The final urination process is mediated by peripheral nerves. Sympathetic preganglionic neurons pass through the hypogastric plexus and innervate the bladder dome, bladder neck, and urethra. At the same time, they also form synapses with postganglionic parasympathetic neurons such as the pelvic ganglion, which can affect the outflow of parasympathetic nerves. The parasympathetic nerve innervates the bladder through the pelvic nerve. In addition to the above structures, the motor neurons in Onuf’s nucleus on the ventral side of the S1-S2 segment of the sacral cord emit fibers to form the pudendal nerve, which innervates the urethral sphincter and mediates its contraction [77,78,83].

#### 2.2.2. Neurodegenerative changes and corresponding clinical manifestations in MSA

Nerve injury at different sites may lead to different LUT dysfunctions. Abnormal lower urinary tract function is usually caused by involuntary contraction of the detrusor caused by bladder filling, a loss of the voluntary micturition reflex and severe urethral dysfunction [84]. MSA lesions extensively involve the central and peripheral nerves that control the lower urinary tract. The pathological changes in the hypothalamus, substantia nigra, locus coeruleus, pons medulla raphe and vermis cerebelli are the basis of overactive bladder (OAB) symptoms. In addition, the loss of the direct effect of the basal ganglia on the PMC and the change in the D1 dopaminergic pathway in the basal ganglia of the frontal cortex are also involved in the occurrence of OAB [76,78]. Meanwhile, the loss of tyrosine hydroxylase neurons in the PAG may lead to the disinhibition or overactivation of the PMC, resulting in excessive detrusor activity [26,85]. The pathological changes in the pontine continence center (PCC) lead to an inability to store urine, a decrease in bladder capacity and premature excretion of urine [86]. PMC lesions impair detrusor contraction and the inhibitory effect of this region on neurons in the L region of Onuf’s nucleus, leading to neurogenic bladder dysfunction, including detrusor hyperreflexia and weakness, as well as urethral sphincter weakness. The clinical manifestations are frequency, urgency, incontinence and urine retention [77,85]. Among them, urgency and frequency of urination are very common at the early stage of the disease, usually in the first year after onset [87]. In addition, the PMC is vital to coordinate detrusor activation and sphincter inhibition. Therefore, pathological changes in the PMC will lead to simultaneous contraction of the detrusor and sphincter during urination, which is called ‘detrusor sphincter dyssynergia’ [83].

In the periphery, the loss of preganglionic parasympathetic neurons and the motor neurons of Onuf’s nucleus at spinal cord levels S2–S4 is related to significant urinary dysfunction in MSA. The former leads to impaired bladder contractility, resulting in incomplete bladder emptying. The latter leads to the denervation of the external urethral sphincter and urinary incontinence [88]. Seventy percent of MSA patients will experience a large amount of residual urine after bladder emptying and the use of catheters after an average of 4 years [89,90]. In addition, the open bladder neck at the beginning of bladder filling is common in MSA, which may be related to sympathetic nerve ­involvement [81].

In addition, in repeated urodynamic tests of MSA patients, there is a tendency for detrusor overactivity caused by central dysfunction to transition to detrusor low compliance caused by preganglionic dysfunction, followed by cholinergic hypersensitivity detrusor contraction. During the disease course of these patients, the lesion site leading to detrusor dysfunction seems to change from the center to the periphery [91,92]. The discovery of a-synuclein in the nerve terminals of the detrusor and external urethral sphincter in patients with multiple systems also supports the important role of lower urinary peripheral neuropathy in urinary dysfunction [93]. A clinical research evaluated the effect of gender on urinary symptoms the prevalence of urinary symptoms was similar in male and female patients, incontinence was more common in females [94]. To differentiate MSA from PD in the early stage, a retrospective study analyzed the utility of urodynamic study parameters, including postvoid residuals (PVR), detrusor overactivity (DO), degree of bladder contraction, and mean duration of motor unit potentials (MUPs) in EAS-EMG. The results showed that PVR > 150ml during free-flow study strongly indicated MSA rather than PD [95]. Another clinical study evaluated the differences in urodynamic findings between MSA and PD patients. The results found that MSA patients showed lower maximal flow rate, larger postvoid residual with decreased compliance, and impaired contractility, while detrusor overactivity and associated urine leakage were common in PD [96].

### 2.3. Sexual dysfunction

#### 2.3.1. Normal genital control

Normal male sexual function is inseparable from normal erections and ejaculation. Erections can be divided into three categories according to relevant stimuli, including psychological erections caused by audio-visual stimuli, reflex erections caused by somatosensory stimuli, and penis swelling at night related to REM sleep. Among the 3 types of erections, reflex erections require a complete sacral cord, especially the IML cell column [97–99]. Conversely, adrenergic neurons mediate the contraction of the penile artery and trabecular smooth muscle, leading to penile detumescence. In addition to adrenergic fibers, many other substances can also mediate penis detumescence. Prostaglandin H2, prostaglandin F2α, thromboxane A2 and other contractile prostaglandins can attenuate the expansion of NO. Angiotensin II may cause the contraction of the cavernous body through the AT-1 subtype receptor [100]. In summary, the participation of the sacral parasympathetic nerve, the thoracolumbar sympathetic nerve and the somatic nerve (pudendal nerve) is required for the normal erection and detumescence of the penis. Sympathetic and parasympathetic nerves converge from neurons in the spinal cord and peripheral ganglia to form cavernous nerves, affecting the neurovascular events required for swelling and detumescence. The pudendal nerve originates from Onuf’s nucleus and innervates the muscles of the ischial cavernous body and bulbar cavernous body, leading to the contraction required during the rigid erection phase [101].

**Figure 3.**
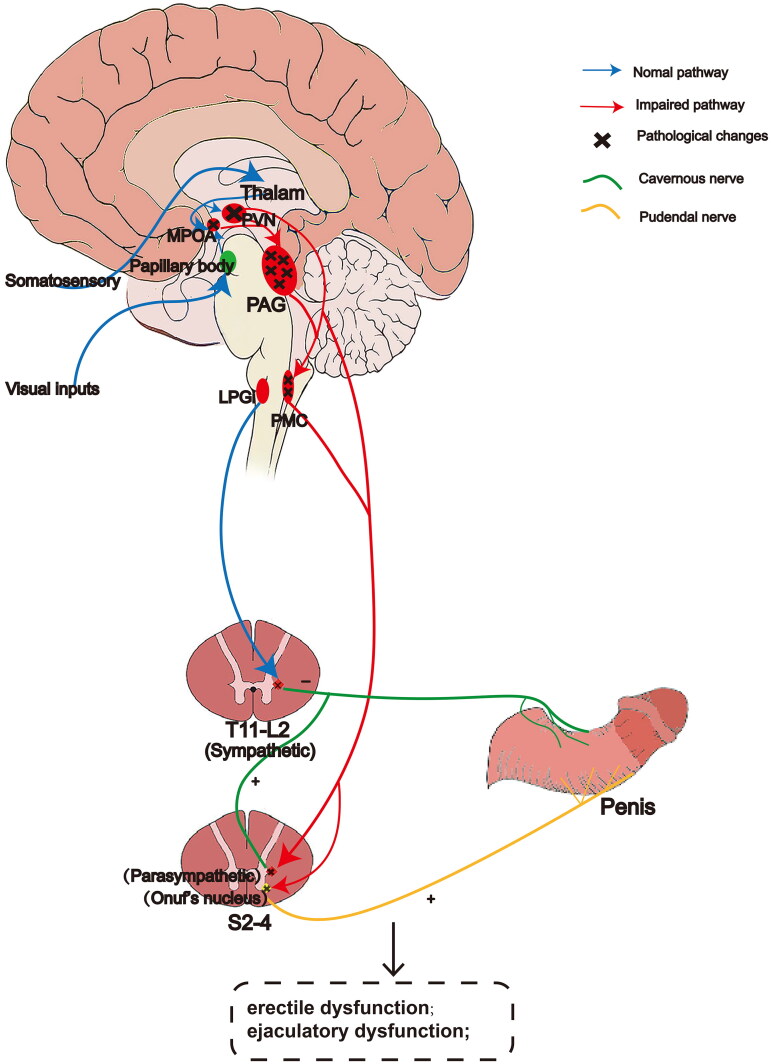
A schematic diagram of the main neural circuits related to erectile function control in MSA. Libido and erection are proved to be regulated by the hypothalamus, especially in MPOA and PVN. The somatosensory input of genitalia rises in the anterior part of the spinal cord and projects to MPOA/PVN through the thalamic nucleus. Sexual visual input from the retina reaches MPOA through the papillary body. Oxytocinergic neurons in hypothalamic PVN can promote erection by projecting directly to the sacral spinal cord, PAG and PMC in the midbrain. Stimulation of the medullary reticular structure, especially LPGi, can activate the sympathetic fibers of the pudendal nerve and promote detumescence. In the periphery, autonomic and somatic nerves (sacral parasympathetic nerves, thoracolumbar sympathetic nerves and somatic nerves) are involved in the tumescence and detumescence of penile erection. PVN: paraventricular nucleus; MPOA: medial preoptic area; PAG: periaqueductal gray area; PMC: pontine micturition center; LPGi: lateralis paragigantocellularis nucleus; T: thoracic; L: lumbar; S: sacral, +: tumescence, −: detumescence.

In addition, the central nervous system also plays an important role in the control of penis erection and detumescence. The medial preoptic area of the hypothalamus (MPOA) and PVN are considered the most important regions for regulating sexual desire and erection. Somatosensory input from the genitals is projected to the MPOA/PVN through the thalamus, while pornographic visual input from the retina arrives at the MPOA through the papillary body. Oxytocin neurons in the PVN are believed to promote erection by projecting to the midbrain PAG and PMC and directly projecting to the sacral cord [97]. In addition, slight stimulation of the medullary reticular formation, especially the lateralis paragigantocellularis nucleus (LPGi), can activate sympathetic nerve fibers passing through the pudendal nerve in anesthetized rats [102]. In summary, these preganglionic neurons in the medulla, pons and diencephalon project to the spinal sympathetic nerve, parasympathetic nerve and pudendal motor neurons, participating in the control of the penile erection and detumescence erection. A brief schematic of the neural control of erection is shown in Figure 3.

Compared with male genitalia, research on female genitalia is limited at present. Female sexual arousal is a kind of vascular congestion and neuromuscular event. During sexual arousal, the blood flow to the uterus, vagina, clitoris and labia minora increases, leading to increased uterine and Bartholin’s gland secretion, glans clitoris protrusion, and labia minora ectropion and congestion [103]. These changes occur through the innervating pelvic and hypogastric nerves. In addition, the hypothalamus plays a crucial role in regulating female sexual behavior. The oxytocin neurons in the ventromedial hypothalamus and MPOA have been proven to directly project to the sacral cord, thereby promoting sexual arousal and the protrusion of the vagina and clitoris [97,104].

#### 2.3.2. Neurodegenerative changes and corresponding clinical manifestations in MSA

In MSA, sexual dysfunction is caused by the interruption of the central autonomous network, among which erectile dysfunction (ED) is the earliest and most common symptom. The involvement of the hypothalamus in MSA leads to its impaired role in promoting sexual desire and erection. In addition, the dorsal vagal nucleus, the IML of the spinal cord and Onuf’s nucleus are generally involved, thereby affecting the sympathetic, parasympathetic and somatic inputs to the genitals, resulting in erectile and ejaculatory dysfunction in MSA. In addition to neurodegenerative diseases, the ED in MSA may also be caused by several other disease-related factors, such as psychosocial stress, fatigue, difficulty in performing fine finger movements, and low self-esteem associated with increased loss of independence [99,105]. For women with MSA, the main manifestations of sexual dysfunction are vaginal dryness, decreased sexual desire and difficulty in reaching orgasm [90]. In addition, the severity of sexual dysfunction increases with the duration of the disease [106].

### 2.4. Gastrointestinal dysfunction

#### 2.4.1. Normal gastrointestinal control

Gastrointestinal function is mainly driven by parasympathetic vagal output and is antagonized or inhibited by sympathetic nerves [107]. The parasympathetic preganglionic inputs of the abdominal organs and the esophagus and gastrointestinal tract above the splenic flexure of the colon come from the dorsal nucleus of the vagus nerve, while the extraintestinal innervation of the digestive tract (descending colon, sigmoid colon and rectum) below the splenic flexure comes from the parasympathetic pelvic nerves of the presacral ganglion (S2–S3 medial lateral cell column) [90,108]. In addition, the most important system for regulating the lower gastrointestinal peristalsis reflex, the enteric nervous system (ENS), is also affected by vagal efferents. Cholinergic efferents in the ENS mediate gastrointestinal excitation by activating serotonin 5HT4 receptors, while the activation of dopamine D2 receptors mediates inhibition [97].

**Figure 4.**
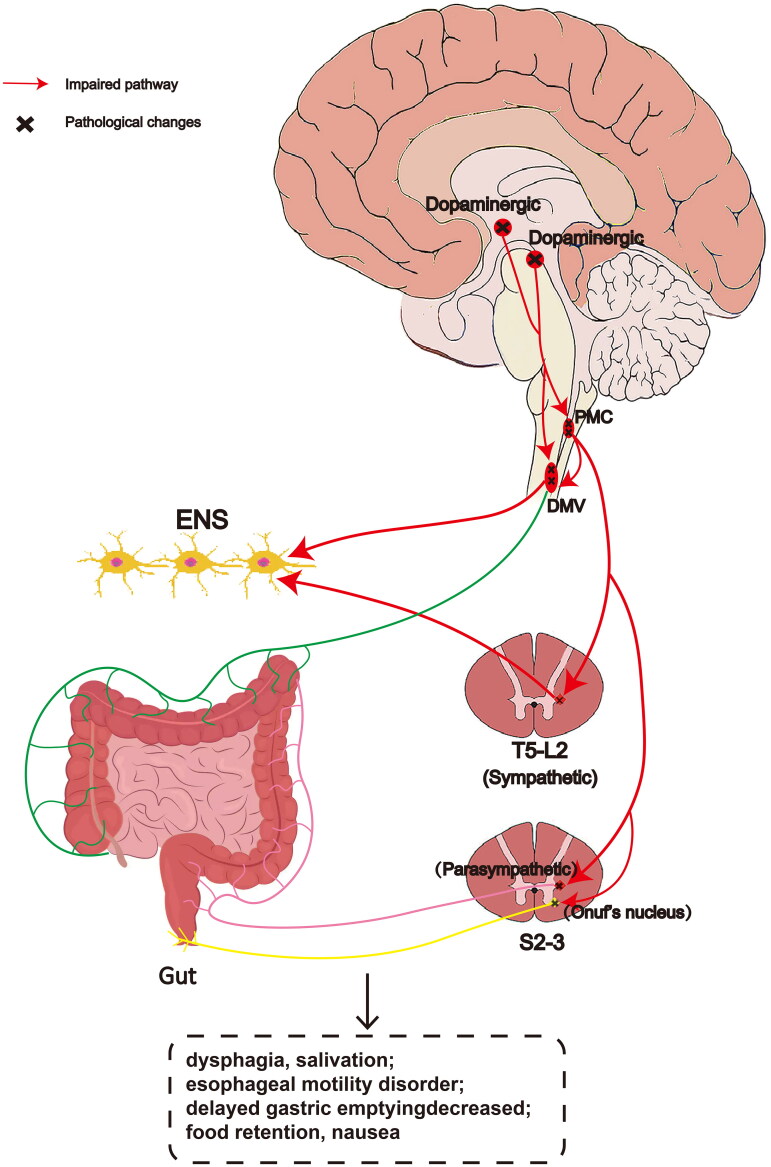
Schematic diagram of neural circuits related to the gastrointestinal tract in MSA. Gastrointestinal tract function is mainly driven by parasympathetic vagal output and modified by sympathetic antagonists. Parasympathetic nerve is divided into cranial nerve (vagus nerve) and sacral nerve (pelvic nerve S2–4), and sympathetic innervation originates from thoracolumbar outflow tract (T5–L2). DMV provides efferent parasympathetic preganglionic fibers for the digestive tract from the esophagus to above the splenic flexure of the Colon, which is connected with ENS neurons to jointly regulate the function of the digestive tract. The parenteral innervation of the digestive tract (descending Colon, sigmoid Colon and rectum) below the splenic flexure comes from the parasympathetic pelvic nerve of presacral ganglion neurons (S2–3 medial lateral cell column). The input of DMV to the whole sacrum and Colon is provided through the spinal cord. Hypothalamic/midbrain dopaminergic cells project fibers to the PMC and DMV in order to control gastrointestinal movement. In addition, gastrointestinal function is also regulated by PAG, cerebellum, thalamus, basal ganglia, anterior cingulate gyrus, insular lobe and prefrontal cortex. The pathological changes eventually lead to gastrointestinal dysfunction. PMC: pontine micturition center; DMV: dorsal motor nucleus of the vagus; ENS: enteric nervous system; PAG: periaqueductal gray area; T: thoracic; L: lumbar; S: sacral.

In addition to the above neural structures, many central structures are also involved in gastrointestinal control. The DMV region targeted by the Barrington nucleus and corticotropin releasing hormone projects to the cecum and the colon, with the exception of the rectum. Therefore, the Barrington nucleus may affect the movement of the whole colon by providing input to the DMV and lumbosacral spinal cord and may participate in gastrointestinal or behavioral control in the stress response [109]. Hypothalamic and midbrain dopaminergic cells also project fibers to the Barrington nucleus and DMV. Moreover, the basal ganglia has been shown to regulate the intestinal movement of experimental animals. In addition to the above areas, gastrointestinal function is also regulated by the PAG, cerebellum, anterior cingulate cortex, insular cortex and prefrontal cortex [108]. A brief schematic of the neural control of the gastrointestinal tract is shown in Figure 4.

#### 2.4.2. Neurodegenerative changes and corresponding clinical manifestations in MSA

In MSA, gastrointestinal dysfunction may be related to the loss of neurons in the hypothalamus, Barrington nucleus, basal ganglia, DMV, and thoracolumbar intermediolateral cell column as well as Onuf’s nucleus [110]. The involvement of the NTS, mesolimbic cholinergic neurons and pre-Bötzinger complex leads to abnormalities of the oropharynx, vocal cords and esophagus, resulting in dysphagia, esophageal motility disorder and delayed gastric emptying [90]. Early severe dysphagia is specific to MSA and is usually very troublesome with the progression of the disease and may lead to fatal inhalation pneumonia [111]. A retrospective study with 297 MSA patients evaluated symptomatic dysphagia within 3 years of onset and quantified dysphagia severity and results indicated that symptomatic dysphagia within 3 years of onset predicted shorter survival in MSA-C and MSA-P patients [112]. A comparative study of swallowing function in patients with MSA and PSP and PD found that dysphagia appeared earlier in the PSP and MSA groups compared to the PD group [113]. A recent clinical study assessed dysphagia in MSA and PD patients and found that patients with MSA predominantly showed more symptoms of oral-phase disturbance and less pharyngeal-phase symptoms, while in patients with Parkinson’s disease, the results were reversed [114].

In addition, a reduction in the efficiency and frequency of swallowing can lead to excessive saliva in MSA patients [90]. Salivation will cause social embarrassment, and more seriously, there will be a risk of asphyxia caused by inhalation and static pneumonia [115]. Esophageal motility disorder can lead to nonperistaltic swallowing, burping, segmental spasms, esophageal dilatation or gastroesophageal reflux. Delayed gastric emptying can lead to food retention, nausea, early satiety and abdominal distension [90]. In addition, serotonergic inputs from the RPa and ROb to the dorsal vagal complex can enhance the activation of vagal motor neurons. The loss of these inputs may also be involved [116].

The degeneration of parasympathetic efferent fibers that regulate the contractility of colonic muscles may be the basis for the slow transmission of colonic contents and lead to a reduction in defecation frequency [108,117]. Abnormalities in pelvic floor skeletal muscles and the anal sphincter caused by central nervous system disorders may change defecation control, resulting in weak tension during defecation and abnormal anal sphincter contraction [97,118]. Complications associated with MSA constipation include intestinal pseudo-obstruction, megacolon, colonic volvulus, fecal impaction, and overflow diarrhea [90]. In addition, the involvement of Onuf’s nucleus in the sacrum is the cause of early fecal incontinence in MSA [108].

Neurodegenerative diseases are characterized by overlapping and comorbidity. A study screened Lewy body disease (LBD) pathology in 230 MSA autopsy patients, and compared the clinical and genetic characteristics of MSA+LBD with or without LBD and 652 LBD patients [119]. The results found that LBD was observed in 11 patients with MSA, which may be related to genetic risk factors. A recent study found that 12 cases of MSA patients had abundant neuronal cytoplasmic inclusions, glial hyperplasia of the hippocampus and severe neuronal loss of medial temporal lobe atrophy in 146 MSA autopsy patients [120]. Compared with typical MSA, these MSA patients have a longer course of disease and a higher prevalence of cognitive impairment, but they lack the clinical characteristics of frontotemporal lobar degeneration-synuclein. Although there were differences in clinical manifestations, they shared common pathological features, which indicated that a subgroup of MSA may be easily affected by marginal structures. MSA is a clinically and pathologically heterogeneous neurodegenerative disease, which may be related to distinct α-syn strain and the seeding activities, distinct conformation of α-syn, the interaction between α-syn and lipid membranes (such as mitochondria, lysosomes, synaptic vesicles) [121–126].

## 3. Conclusion

Cardiovascular function, sexual function, urine storage and urination, gastrointestinal secretion and movement control mechanisms involve highly interconnected neuronal networks with different neurochemistry properties in different regions of the cortex, diencephalon, brainstem and spinal cord. The deposition of α-syn in these neural network structures leads to early onset and extensive autonomic dysfunction in MSA. These clinicopathological correlations emphasize the importance of integrating information obtained from histopathological studies and animal models with the evaluation and management of clinical manifestations. On the one hand, histopathological studies and animal models provide more effective evaluation methods and treatment targets for clinical manifestations. On the other hand, the evaluation and management of clinical manifestations also provide a more in-depth research direction for the pathogenesis and histopathological research of diseases. Unfortunately, the source of α-syn and the selective vulnerability mechanism of autonomic neural networks in MSA are still unclear, so there are no neuroprotective or neurorepair therapies that can be used to prevent or reverse the progression of MSA. There is an urgent need for further and deeper studies to elucidate the pathogenesis and progression of MSA.

## Data Availability

Data sharing is not applicable to this article as no new data were created or analyzed in this study. This article is a narrative review, not a study.
